# Topical tretinoin alters skin microbiota in patients with mild acne

**DOI:** 10.1016/j.jdin.2023.09.004

**Published:** 2023-09-24

**Authors:** Rebeca Martinez, Omkar Mayur, Kyla Pagani, Danitza Lukac, Jean S. McGee

**Affiliations:** a Department of Dermatology, Beth Israel Deaconess Medical Center, Boston, Massachusetts; b University of Massachusetts Chan Medical School, Worcester, Massachusetts; c Department of Internal Medicine, Lahey Hospital & Medical Center, Burlington, Massachusetts; d Harvard Medical School, Boston, Massachusetts

**Keywords:** acne, *Kingella*, skin microbiome, skin microbiota, therapeutic response, tretinoin

*To the Editor:* There is increasing evidence of the role that the skin microbiota plays in the pathogenesis of acne vulgaris.^1^ We hypothesize that topical tretinoin without known antibacterial properties can induce alteration of the skin microbiome in patients with acne. We conducted a study to assess the changes in the skin microbiota associated with therapeutic response in patients with acne, who were treated with tretinoin 0.05% lotion, Altreno (Ortho Dermatologics).

We prospectively recruited subjects who met our inclusion/exclusion criteria. For visit 1, we swabbed 1 cheek of all subjects to assess their baseline skin microbiomes. For acne patients, we repeated the skin microbiome collection after they underwent treatment with tretinoin for ∼90 days (visit 2) and after they discontinued tretinoin for ∼60 days (visit 3). We also graded their acne severity using the Physician Global Assessment scoring system.^2^ For control subjects, we repeated the skin microbiome collection after ∼90 days (visit 2) without any therapeutic intervention to serve as an internal control; therefore, additional time point (visit 3) was not necessary. The skin swab samples were sent to Diversigen, Inc for downstream processing and analysis (Supplementary Materials, available via Mendeley at https://doi.org/10.17632/kv56sxty73.1).

We recruited 12 control subjects and 12 acne patients; 4 control subjects and 4 acne patients were lost to follow-up after visit 1 (Table I). At baseline, relative abundance of *Kingella* at the genus level was 5.8-fold higher in acne patients compared to that in healthy controls (visit 1) (Fig 1). Then, after treatment with tretinoin, there was no statistically significant difference in the relative abundance of *Kingella* between acne patients and healthy controls (visit 2) (Fig 1). Finally, after discontinuation with tretinoin, there was no statistically significant difference in the relative abundance of *Kingella* in patients with acne between visit 2 and visit 3 (Fig 1). The *P* value was set at <.15. X. We found 3additional taxa at the family level to be significant (Supplementary Results, available via Mendeley at https://doi.org/10.17632/kv56sxty73.1).

**Table I:** Acne status, demographic factors, and Physician Global Assessment scores of study subjects

Acne status	Age	Sex	Race, ethnicity	PGA (visit 1)	PGA (visit 2)	PGA (visit 3)
Control	32	F	White, non-Hispanic	#		
Control	32	M	White, non-Hispanic			
Control	25	M	White, non-Hispanic			
Control	38	F	White, non-Hispanic		#	
Control	27	M	White, non-Hispanic		X	
Control	36	M	White, non-Hispanic		X	
Control	33	F	White, non-Hispanic	#	#	
Control	36	F	White, non-Hispanic			
Control	24	F	White, Hispanic		X	
Control	37	M	White, non-Hispanic			
Control	27	F	White, non-Hispanic		X	
Control	35	M	White, non-Hispanic			
Acne	31	F	Asian, non-Hispanic	2#	0	0#
Acne	38	F	White, non-Hispanic	1	1	0
Acne	27	F	Unknown, Hispanic	2	1	1
Acne	24	M	White, non-Hispanic	1	X	X
Acne	28	F	White, non-Hispanic	2	X	X
Acne	22	F	White, non-Hispanic	1	0	3
Acne	40	F	White, non-Hispanic	1	X	X
Acne	23	F	White, non-Hispanic	1	0	1
Acne	36	F	White, non-Hispanic	2	2	2
Acne	22	F	White, non-Hispanic	3	3	3
Acne	27	F	African, non-Hispanic	2	X	X
Acne	24	F	White, non-Hispanic	2	1	1

PGA score improved in 5 out of 8 patients with acne after treatment with tretinoin (visit 2). PGA score remained the same or further improved in 6 out of 8 patients after discontinuation of tretinoin (visit 3). X: Subjects were lost to follow-up; #: Due to insufficient DNA quantity, failed QC for downstream sequencing/analysis.

*PGA*, Physician Global Assessment; *QC*, quality control.

**Fig 1:**
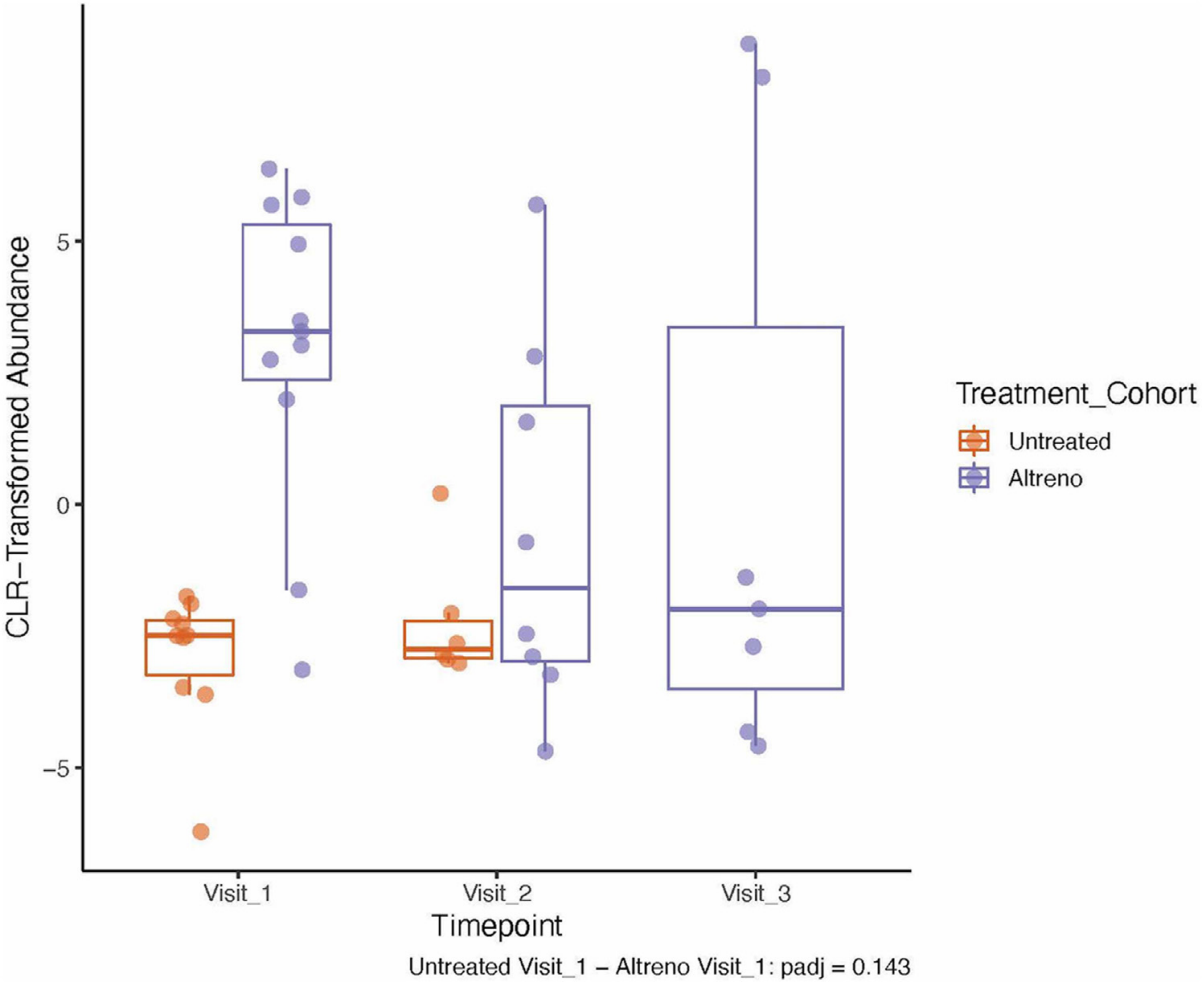
Relative abundance of *Kingella* in untreated vs Altreno. The relative abundance was represented on the Y-axis as a centered log-ratio transformation of the read count of *Kingella* over the read counts of all other taxa within each sample. On visit 1, the median of centered log-ratio–transformed abundances of *Kingella* in patients with acne (ie, Altreno) was 5.8-fold higher than the median in healthy controls (ie, untreated), demonstrating that there is a higher skin burden of *Kingella* on patients with acne at baseline. On visit 2, there was no statistically significant difference between the relative abundances of *Kingella* between patients with acne who were treated with topical tretinoin, Altreno, for approximately 90 days and healthy controls without any therapeutic intervention. On visit 3, after patients with acne discontinued the tretinoin therapy for approximately 60 days, the relative abundance of *Kingella* of patients with acne was unchanged from that of patients with acne on visit 2. *CLR*, Centered log-ratio; *padj*, adjusted *P*-value.

Our study was limited by a small sample size and a high dropout rate, and may have failed to find any small, yet significant, differences in the alpha and beta diversity measures (Supplementary Materials, available via Mendeley at https://doi.org/10.17632/kv56sxty73.1). Additionally, our acne cohort was homogenous, mainly of young female subjects with mild acne amenable to topical therapy only. Therefore, our study findings may not translate to a broader population. Despite these limitations, we observed that the acne patients at baseline had a higher skin burden of *Kingella* compared to the control subjects. With tretinoin treatment in patients with acne, the relative abundance of *Kingella* on the skin normalized to the level similar to the control subjects without acne. Moreover, the therapeutic effect of tretinoin on the skin microbiota (ie, *Kingella*) was maintained for 60 days despite discontinuation of its use. Several well-known species of *Kingella* are pathogenic; however, recent studies have shown that *Kingella* at the genus level is found in the healthy skin microbiome.^3^^,^^4^ Overall, our findings demonstrate that topical application of tretinoin may alter the skin microbiome in patients with acne, suggesting a potential additional therapeutic mechanism of tretinoin.

## Conflicts of interest

None disclosed.
